# Robotic surgery versus conventional laparoscopy in sigmoid colectomy for diverticular disease-a comparison of operative trauma and cost-effectiveness: retrospective, single-center analysis

**DOI:** 10.1007/s00423-024-03382-0

**Published:** 2024-06-27

**Authors:** Jaroslav Presl, M. Ehgartner, L. Schabl, F. Singhartinger, A. Gantschnigg, E. Wallner, T. Jäger, K. Emmanuel, H. Kessler, O. O. Koch

**Affiliations:** 1 Department of Visceral and Thoracic Surgery, Paracelsus Medical University, Salzburg, Austria; 2 Department of Colorectal Surgery, Cleveland Clinic, Cleveland, OH USA

**Keywords:** Sigmoid colectomy, Diverticular disease, Robotic surgery, Miniinvasive surgery, C-reactive protein, Hemoglobin drift

## Abstract

**Purpose:**

Robotic assisted surgery is an alternative, fast evolving technique for performing colorectal surgery. The primary aim of this single center analysis is to compare elective laparoscopic and robotic sigmoid colectomies for diverticular disease on the extent of operative trauma and the costs.

**Methods:**

Retrospective analysis from our prospective clinical database to identify all consecutive patients aged ≥ 18 years who underwent elective minimally invasive left sided colectomy for diverticular disease from January 2016 until December 2020 at our tertiary referral institution.

**Results:**

In total, 83 patients (31 female and 52 male) with sigmoid diverticulitis underwent elective minimally invasive sigmoid colectomy, of which 42 underwent conventional laparoscopic surgery (LS) and 41 robotic assisted surgery (RS). The mean C-reactive protein difference between the preoperative and postoperative value was significantly lower in the robotic assisted group (4,03 mg/dL) than in the laparoscopic group (7.32 mg/dL) (*p* = 0.030). Similarly, the robotic´s hemoglobin difference was significantly lower (*p* = 0.039). The first postoperative bowel movement in the LS group occurred after a mean of 2.19 days, later than after a mean of 1.63 days in the RS group (*p* = 0.011). An overview of overall charge revealed significantly lower total costs per operation and postoperative hospital stay for the robotic approach, 6058 € vs. 6142 € (*p* = 0,014) not including the acquisition and maintenance costs for both systems.

**Conclusion:**

Robotic colon resection for diverticular disease is cost-effective and delivers reduced intraoperative trauma with significantly lower postoperative C-reactive protein and hemoglobin drift compared to conventional laparoscopy.

## Introduction

The prevalence of left colon diverticular disease increases with age, especially in industrialized countries [1, 2]. While the prevalence is still around 10% in people under 40 years of age, it rises to as much as 70% in people over 80 [3].

The left colon is affected in most cases in Western countries [4]. 10 to 25% of the affected patients develop diverticulitis, which is associated with complications in about 12% [5, 6].

Surgery represents a part of the therapeutic regime of the above diseases [7–9]. The optimal outcome of surgery consists of a cure of the underlying disease and a procedure that is as gentle as possible for the patient. Over the last decades, minimally invasive colorectal surgery in the form of laparoscopic resection was established. It has shown shorter hospital stays, lower complication rates, and less postoperative pain than conventional open surgery [10–16].

However, the restricted field of view and instrument movement, less intuitive working posture missing ergonomics, especially in high BMI patients, have been described as possible downsides to laparoscopy [17, 18].

As a solution, robotic surgical telemanipulators have become increasingly established in various surgical fields since the late 1990s [19–21]. Robotic assisted interventions in colorectal surgery for benign disease, especially in patients with diverticular disease, have become a topic of discussion in recent years. More extensive studies using national databases were published, showing controversial results; a lower conversion rate [22–24], a shorter hospital stay [24] and longer operative time [22–25] in the robotic groups. Only Ogilvi Jr. et al. compared 2019 the costs of both minimally invasive procedures and showed significantly increased total hospital charges for the robotic procedure [26].

The primary aim of this study is to compare elective laparoscopic and robotic left-sided sigmoid colectomies for diverticular disease on the extent of operative trauma and the costs, which are the most outspoken controversy in the discussion of both procedures.

The secondary aim is to compare the operative time, conversion rate, length of postoperative hospital stay, and postoperative morbidity.

## Patients and methods

The study was approved by the ethic committee of Land Salzburg EK Nr: 1055/2023. Data were retrieved from our prospective clinical database. All consecutive patients aged ≥ 18 years who underwent elective minimally invasive left sided colectomy for diverticular disease from January 2016 until December 2020 were included. Two retrospective cohorts were built, including patients undergoing conventional laparoscopic surgery (LS) and robotic assisted surgery (RS). Exclusion criteria were an open and emergency surgery.

The following data has been collected to compare the groups. Demographic data (age, sex, BMI and ASA) and length of postoperative hospital stay (LOS). To stratify the severity of diverticular disease, the German diverticular disease classification (CDD) was adopted [27]. The intraoperative conversion rate, postoperative complications classified according to Clavien – Dindo, 30-days and 90-days mortalities, length of the resected colon, the difference of preoperative and postoperative C-reactive protein (CRP) in mg/dL, hemoglobin in g/dL, the first flatus and the first oral intake were examined. The comparison of costs was built upon the costs of material and staff per surgical procedure and per day of postoperative hospital stay.

LS and RS, were conducted in a 22-degree Trendelenburg lithotomy and 12-degree right lateral position.

For laparoscopy, one 10 mm supraumbilical trocar, one 12 mm trocar in the right lower abdomen, one 5 mm trocar in the right middle abdomen, and one 7 mm Air-Seal® trocar in the left middle abdomen are standardly used.

The robotic operations were done using the Da Vinci Robotic Surgical X^™^ System (Intuitive Surgical® System, Sunnyvale, CA, USA) until November 2020. Since then, the Xi^™^ System has been adopted. In both systems, we used five trocars, three 8 mm, and one 12 mm robotic trocars and one 7 mm Air-Seal® trocar.

In both groups, a tubular sigmoid resection with sparing of the inferior mesenteric artery (IMA) and superior rectal artery (SRA) followed by an end-to-end intracorporeal anastomosis with a circular 28 mm EEA^™^ Circular Stapler (Medtronic®, Dublin, Irland) were performed. The resected bowel segment was retrieved exclusively via Pfannenstiel incision in all cases.

The splenic flexure was either partially mobilized from lateral, or completely taken down in a medio-lateral approach, which includes complete separation of the transverse mesocolon from the pancreas. The decision about the type of flexure mobilization was left to the surgeon.

### Statistical analysis

The statistical analysis was performed using IBM SPSS® version 27 for macOS (IBM, Armonk, New York, United States). The descriptive data were presented as absolute values, relative values in percentages, mean values and their standard deviation. Comparison between the mean values of individual parameters of the respective groups was made using the t-test and the Mann-Whitney U-test, depending on the presence of a normal distribution. Values were tested for normal distribution using the Kolmogorov-Smirnov test. A p-value of < 0.05 was considered statistically significant calculating differences.

## Results

In total, 83 patients (31 female and 52 male) with sigmoid colon diverticulitis underwent elective minimally invasive surgery, of which 42 underwent conventional laparoscopic surgery (LS) performed by 6 surgeons and 41 robotic assisted surgery (RS) by 3 surgeons. There were no differences between the two groups according to mean age, BMI, and ASA classification at the time of surgery (Table 1).

In terms of Classification of Diverticular Disease (CDD), stage IIB (33.33%) was the most common in laparoscopically operated patients, followed by stage IIA (26.19%) and stage IIIC (23.81%). Among robotic assisted operated patients, stage IIA dominated with 39.02%, followed by stage IIB and IIIB with 31.71% and 24.39%, respectively. (Table 2)

No significant difference was found in the mean operating time (incision to suture time) comparing RS versus LS with 191.78 vs. 177.43 min (*p* = 0.092). The postoperative length of stay was shorter, with 6.41 days in the RS group compared to 8.48 days in the LS group (p = of 0.017) (Table 3).

The number and severity of complications according to the Clavien-Dindo classification did not differ significantly between both groups [28]. The breakdown of the complications according to the Clavien-Dindo classification can be found in Table 4. In the group of laparoscopic interventions, intraoperative injury of the ureter occurred in one case. There was no conversion to open surgery in any of the groups. During the 30-day and 90-day follow-ups, no deaths were associated with the interventions in either group.

The groups presented a difference in the length of the resected bowel segment, with a mean length of 16.04 cm resected in the LS group and 14.31 cm in the RS group (*p* = 0.005). The robotic assisted group had a significantly lower mean value of 4.03 mg/dl comparing the preoperative and postoperative CRP than the laparoscopic group with a mean of 7.32 mg/dl (*p* = 0.030). Similarly, the Hb difference (hemoglobin drift) was significantly lower in the robotic assisted patients (*p* = 0.039). The first postoperative bowel movement occurred after a mean of 2.19 days in the LS group, and after a mean of 1.63 days in the RS group (*p* = 0.011). The first postoperative solid food intake occurred with no significant difference (Table 5).

An overview of the current overall charge, including the material used for the individual operation, staff costs per mean operation duration as well as mean postoperative hospital stay, revealed lower total costs per operation and postoperative hospital stay for the robotic approach 6058 € vs. 6142 € (*p* = 0.014) as well as lower costs per postoperative hospital stay 2787 ± 1608 € vs. 2109 ± 345 € (*p* = 0.017). The staff costs per procedure remain the same in both groups (Table 6). The material costs analysis for each procedure revealed higher costs for the robotic surgery (2657 € vs. 2160 €). Calculation of the potential acquisition and maintenance cost of the robotic and laparoscopic operation system, respectively, was performed. The costs were calculated on the basis of our institute’s internal calculation bases with a net service life of 10 years and 1000 h per year (50% of the gross calculation with 250 days per year, 8 h per day) for both the laparoscopic tower system and the robotic system. With a purchase price of over 2 million Euros, the DaVinci Robotic Xi™ System is more expensive than a laparoscopy tower system (Table 7).

## Discussion

The surgical management of diverticular disease has continued to evolve over the past years, as did the operative technique. Many studies have shown a clear superiority of the laparoscopic approach to open surgery [10–16]. This trend continues as robotic surgery is adopted for colon resection for diverticular disease.

In this study, we could demonstrate that robotic surgery is safe regarding perioperative morbidity compared to conventional laparoscopic sigmoid colon resection for diverticular disease. Moreover, our results showed the superiority of the robotic approach due to shorter postoperative hospital stay, reduced intraoperative trauma with significantly lower postoperative CRP and hemoglobin drift, and faster recovery of the bowel movement.

We had no conversion in any of the groups, which should be discussed as a potential selection bias at the beginning of the robotic program. To contradict that, 70% of patients in the robotic and 59% in the laparoscopic group had an abscess at the time of diagnosis and were classified as CDD Type IIa or IIb. A positive trend of the conversion rate odds lowering in robotic surgery was also reported in the latest review from Larkins et al. as well as from Maciel V et al. and Raskin E et al. [22, 23, 29] Rashidi et al. showed that not only robotic surgery itself shows a lower conversion rate but also the high volume robotic surgeons have significantly lower conversion rates then high volume laparoscopic surgeons. He defined those as surgeons performing more than 30 colorectal procedures within a year [30]. Bastawrous et al. confirmed the positive influence of high volume (30 or more robotic colorectal cases per surgeon per year) on conversion rate between robotic surgeons only and showed that they even have lower operative times and shorter hospital length of stay [31]. Furthermore, having conventional laparoscopic expercience before starting robotic assisted procedures is thought to be helpful. Even though this has been previously discussed, there is little evidence confirming a real benefit. Flynn et al. found in his systematic review only three operative studies comparing robotic and laparoscopic learning curves. He found no difference in learning curves for low anterior resection but a significant difference for right colectomy. Surprisingly these three operative studies showed a shorter operative times for robotic platform at some point in the study. In Flynn´s review included simulation studies showed greater time advantages for complex tasks, such as knot tying [32]. In Marecik´s et al. study 15 novices performed an anastomosis on a porcine intestinal model. The anastomoses were subjected to pneumatic pressure to quantify the “leak pressure”. He showed significantly higher quality of robotic anastomoses [33]. 

The C-reactive protein, postoperative hemoglobin drift, and postoperative first bowel movement were examined to evaluate operative trauma. CRP and its positive correlation to the extent of operative trauma have already been investigated by Neumaier et al. [34, 35]. The lower CRP after robotic surgery shows that robotic surgery causes less operative trauma. This finding is supported by a lower difference in preoperative to postoperative hemoglobin, the so called “hemoglobin drift” in the robotic study arm. The hemoglobin drift is considered greater after surgical procedures with higher intraoperative IV fluid and blood requirements [36]. Lastly, earlier first bowel movement at postoperative day 1,63 ± 0,73 after robotic surgery compared to laparoscopy at 2,19 ± 0,99 days supports the notion of gentler tissue management during robotic surgery. This finding has also been described in a propensity-matched study by Ogilvie et al. and Beltzer et al. [26, 37]. We explain these results with a higher precision of the dissection in the anatomic plains, during the robotic surgery.

The splenic flexure was either partially mobilized from lateral, which was more often done in RS, or completely taken down in a medio-lateral approach, as performed more often in conventional laparoscopy. This result demonstrates the ability of the robot to complete a challenging lateral mobilization with the EndoWrist technique, whereas conventional laparoscopy advocates the medio-lateral approach.

The mean length of our robotically retrieved specimens (14,31 cm ± 8,12) was shorter than those harvested with conventional laparoscopy. Unfortunately, pathologists measured the specimen lenght after formalin fixation, rather than surgeons directly after retrieval on the back table. Goldstein et al. described a considerable shrinkage of up to 57% of the length of the resected colon within the first few minutes after removal and additionally after formalin fixation [38]. Only few papers study the impact of the specimen length on the recurrence of diverticulitis. Thaler et al. has examined 236 patients and found, using regression analysis, that the specimen length had no impact on the recurrence rate [39].

Few studies have discussed costs associated with using robotics for minimally invasive surgery in the past, with a controversial result [26, 40, 41]. Robotics become increasingly affordable due to permanent price reduction of the robotic instruments and their increased lifetime, ensuring permanent price convergence. In our institution, the material costs for robotic surgery remain about 500€ higher per procedure than for laparoscopy. However, due to the shortage of postoperative hospital stay (*p* = 0,017), we have reduced total costs in favor of robotic procedure (*p* = 0,014)The main reason for this difference is two Clavien-Dindo IIIb complications (one suprapubic wound infection with negative pressure wound therapy and one postoperative intraabdominal bleeding with the need of laparoscopic revision) and one intraoperative complication in the laparoscopic group (ureter lesion) causing much longer postoperative hospital stay with additional costs of about 12,400€.

The strength of this study is data sourcing from a prospectively mantained database of our centre with a highly experienced team of colorectal surgeons. Furthermore, similar baseline characteristics, comparable case dissemination in both groups according to CDD-Classification, and limited inclusion of only the left colon are minimizing selection bias.

Limitations are to be mentioned: (1) The study is retrospective and the numbers of our study were based on the total number of laparoscopic and robotic cases available and not on a power analysis. (2) Our cost analysis does not include the acquisition and maintenance costs for both systems, which are depreciated in different ways over at least 6 years. (3) The LS was performed by 6 colorectal surgeons of which 3 did all robotic assisted procedures. To the beginning of our robotic program the surgeons were chosen based on their interests on robotic surgery and general experience in laparoscopic surgery, not according to the volume of colorectal procedures. (4) The pathologists measured the specimens lenght after the formalin fixation.

## Conclusion

We demonstrate that robotic surgery is cost-effective and safe regarding perioperative morbidity and delivers reduced intraoperative trauma with significantly lower postoperative CRP and hemoglobin drift and faster recovery of the bowel movement compared to conventional laparoscopic sigmoid colon resection for diverticular disease.

This study provides further evidence to a growing body of colorectal research that shows comparable outcomes using a technologically advanced and increasingly affordable platform.

**Table 1:** Gender, Age, BMI & ASA

	Laparoscopic Surgery	Robotic Surgery	*p*-Value
**Patient, n**	*n* = 42	*n* = 41	0.529
**Gender, n (%)**			0.529
w	14 (33.33%)	17 (41.46%)	
m	28 (66.67%)	24 (58.54%)	
**Ø Age (y) ± SD**	58.17 ± 12.34	54.46 ± 12.36	0.176
**Ø BMI ± SD**	27.31 ± 3.89	28.32 ± 4.78	0.293
**Ø ASA ± SD**	2.12 ± 0.59	1.98 ± 0.52	0.337

**Table 2:** CDD-Classification

	Laparoscopic Surgery	Robotic Surgery	*p*-Value
**CDD-Classification**, **n (%)**	42	41	
IIA	11 (26.19%)	16 (39.02%)	0.327
IIB	14 (33.33%)	13 (31.71%)	0.984
IIIA	0	0	
IIIB	7 (16.67%)	10 (24.39%)	0.549
IIIC	10 (23.81%)	2 (4.88%)	0.139

**Table 3:** Operative time and hospital stay

	Laparoscopic Surgery	Robotic Surgery	*p*-Value
**Ø OP-Time (min) ± SD**	177.43 ± 47.04	191.78 ± 68.51	0.092
**Complete mobilization of splenic flexure**	12	7	
**Partial mobilization of splenic flexure**	25	33	
**Ø Postoperative hospital stay (d) ± SD**	8.48 ± 4.89	6.41 ± 1.32	**0.017**

**Table 4:** Complications and conversion rate

	Laparoscopic Surgery	Robotic Surgery	*p*-Value
**Complications, n (%)**	10 (23.81%)	4 (10%)	0.271
Clavien-Dindo I	5 (11.9%)	3 (7.32%)	
	*3x suprapubic hematoma*	*1x suprapubic seroma*	
	*1x superficial suprapubic wound infection*	*1x neurological irritation of L3/L4*	
	*1x suprapubic seroma*	*1x high blood pressure*	
Clavien-Dindo II	0	1 (2.44%)	
		*1x infection with antibiotic therapy*	
Clavien-Dindo IIIa	2 (4.76%)	0	
	*1x anastomotic bleeding*		
	*1x suprapubic abscess 4 weeks after surgery*		
Clavien-Dindo IIIb	2 (4.76%)	0	
	*1x suprapubic wound infection with vacuum therapy*		
	*1x postoperative bleeding with operative revision*		
Intraoperativ	1 (2.38%)	0	
**Conversion to open surgery, n (%)**	0	0	
**30-days-Mortality, n (%)**	0	0	
**90-days-Mortality, n (%)**	0	0	

**Table 5:** Length of specimen, postoperative difference of CRP in mg/dL and Hemoglobin g/dL, postoperative bowel movement

	Laparoscopic surgery	Robotic surgery	*p*-Value
**Ø Length of specimen (cm) ± SD**	16.04 ± 4.33	14.31 ± 8.12	**0.005**
**Ø CRP-difference ± SD**	7.32 ± 7.3 (*n* = 33)	4.03 ± 3.84 (*n* = 31)	**0.030**
**Ø Hb-difference ± SD**	1.89 ± 1.21 (*n* = 37)	1.31 ± 1.14 (*n* = 37)	**0.039**
**Ø First bowel movement (d) ± SD**	2.19 ± 0.99	1.63 ± 0.73	**0.011**
**Ø First postoperative solid food intake (d) ± SD**	1.76 ± 0.88	1.59 ± 0.74	0.447

**Table 6:** Costs in € per procedure and Mean Hospital Stay

	Laparoscopic surgery	Robotic surgery	*p*-Value
**Material costs for Surgery (€)**	**2159.5**	**2656.5**	
Hospital stay costs (€/per day)	328.8	328.8	
Staff costs (€/per Minute)	6.74	6.74	
Mean operation duration	177.43 ± 47.04	191.78 ± 68.51	
**Mean staff costs per operation duration (€)**	**1196 ± 317**	**1293 ± 179**	**0.092**
Mean postoperative hospital stay (days)	8.48 ± 4.89	6.41 ± 1.32	
**Total mean costs per hospital stay (€)**	**2787 ± 1608**	**2109 ± 345**	**0.017**
***Mean Total Costs €***	***6142 ± 1676***	***6058 ± 482***	***0.014***

**Table 7:** Acquisition and maintenance costs statement RS and LS in €

	Laparoscopic surgery	Robotic surgery
Calculated useful life (y)	10	10
Calculated net hours per year	1000	1000
Acquisition costs	2 015 918	114 205
Costs per use	276	13
Costs per year	201 592	11,420
Costs per hour	202	11
Costs per minute	3,36	0,19

## Data Availability

The datasets generated during and/or analysed during the current study are available from the corresponding author on reasonable request.
